# Progressive volume reduction and long-term aneurysmal collapse following flow diversion treatment of giant and symptomatic cerebral aneurysms

**DOI:** 10.3389/fneur.2022.972599

**Published:** 2022-08-11

**Authors:** Kristina Sirakova, Marin Penkov, Svetozar Matanov, Krasimir Minkin, Kristian Ninov, Asen Hadzhiyanev, Vasil Karakostov, Irena Ivanova, Stanimir Sirakov

**Affiliations:** ^1^Radiology Department, Alexandrovska Hospital, Sofia, Bulgaria; ^2^Radiology Department, University Hospital St Ivan Rilski, Sofia, Bulgaria; ^3^Neurosurgery Department, University Hospital St Ivan Rilski, Sofia, Bulgaria; ^4^Clinical Laboratory Department, University Hospital St Ivan Rilski, Sofia, Bulgaria

**Keywords:** aneurysm, flow diversion, shrinkage, embolization, giant

## Abstract

**Background:**

The primary goal of conventional endovascular and microvascular approaches is the clinical and radiological resolution of the symptomatic aneurysm-induced mass effect. This study assessed the volume changes and mass effect reduction due to sac shrinkage after treatment with flow diverter stents (FD) for unruptured cerebral aneurysms.

**Methods:**

We analyzed retrospectively 36 symptomatic aneurysms that were larger or equal to 25 mm in diameter in patients treated at our center from January 2016 to April 2022. Radiological and clinical outcomes were analyzed, including aneurysmal volume changes and resolution of aneurysm-related symptoms.

**Results:**

At 6 months, 25 aneurysms decreased in size, 2 remained unchanged, and 9 aneurysms demonstrated a post-treatment dimensional increase. At 12 months, 30 aneurysms showed a progressive radiological volume reduction. Either no change or negligible shrinkage was observed in the remaining six aneurysms. At 24 months, 32 aneurysms showed aneurysmal shrinkage by a mean 47% volume loss with respect to baseline. At the last follow-up, all 13 patients who had presented with third cranial nerve palsy showed improvements. Complete reversal of the pretreatment edematous changes was confirmed in all cases. The overall post-treatment complication rate was 8.3%, as 3 patients experienced non-fatal delayed rupture of their aneurysm. There was no mortality in this study.

**Conclusion:**

Flow diversion could effectively induce progressive aneurysmal shrinkage and resolution of the mass effect associated with giant symptomatic cerebral aneurysms.

## Introduction

Giant (≥25 mm) intracranial aneurysms are a unique subcategory of cerebral aneurysms with poor natural history and technically demanding treatment options (1, 2).

If left untreated, patients with such aneurysms have a dismal prognosis, with an increased risk of rupture and poor long-term clinical outcomes (3, 4). Beyond the poor prognosis and increased risk of rupture, these aneurysms have other clinical consequences. Peri-aneurysmal edemic changes in adjacent brain structures are typically associated with these aneurysms (5). Depending on their distribution across the cerebral circulation, the functional integrity of some cranial nerves may be compromised (6, 7). Treatment strategies for these uncommon intracranial aneurysms remain a matter of substantial debate, because evidence from large clinical trials remains lacking (8). Therefore, a compelling biological rationale supports early treatment for medically suitable patients.

The microvascular clipping of large or giant cerebral aneurysms carries favorable chances for a definitive and durable cure (9). However, this treatment method still carries substantial risks (10, 11). Primary endovascular coil embolization with or without adjunctive devices has yielded varying angiographic and treatment results (12, 13). More recently, endoluminal flow diverter stents have been associated with high rates of complete aneurysm occlusion and an acceptable safety profile (14, 15). After endovascular flow diversion for large or giant aneurysms, the best possible outcome is significant shrinkage of the sac and complete aneurysmal obliteration (16, 17).

A combined study of long term radiological and clinical data on flow diverter treatment of large or giant symptomatic and unruptured cerebral aneurysms would be valuable but had not been reported. Therefore, this study analyzed the clinical and radiological outcomes of patients treated with flow diversion, and specifically evaluated the treatment effects on aneurysmal sac shrinkage and volume reduction.

## Materials and methods

### Adherence to ethical standards and legal requirements

This retrospective study was approved by the local institutional ethical committee, and was designed and performed in accordance with its policies and guidelines. Patient consent was not required, given the study's retrospective nature and the lack of identifying information.

### Data collection and study design

The strengthening of the reporting of observational studies in epidemiology (STROBE) guidelines were followed to collect and report data (18).

### Study design and population

**Figure 1:**
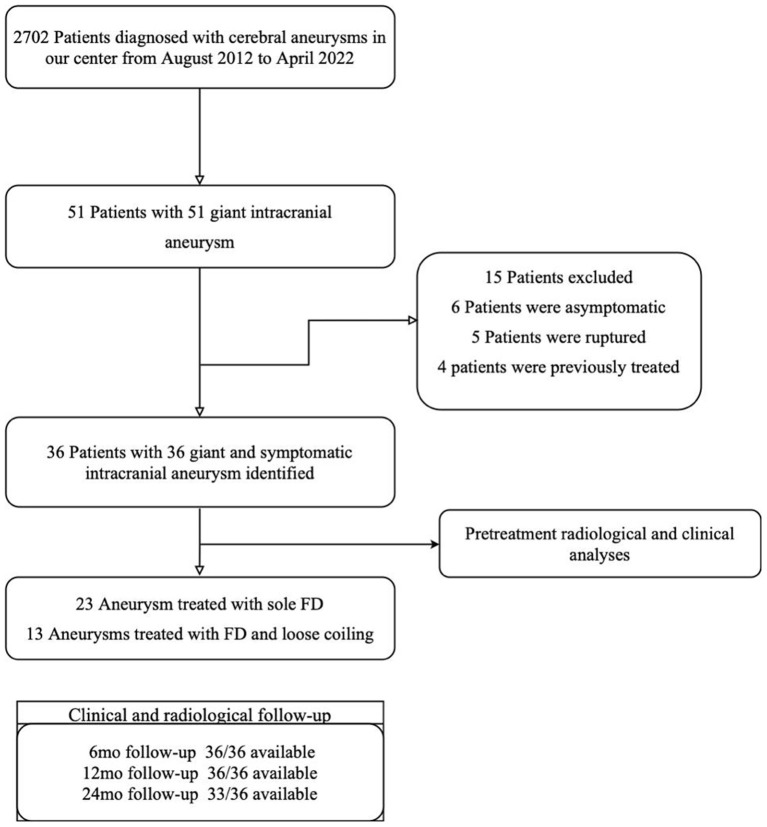
Illustrative flow chart of study design and population.

We examined the local electronic health database by using a unified code (I67.1) from the International Classification of Diseases, ver. 10 to identify patients with unruptured intracranial aneurysms diagnosed and treated in our center. A total of 51 cerebral aneurysms ≥25 mm in diameter were identified. This cohort represented ~1.9% of all aneurysms treated in our center. To evaluate the effect of flow diversion on progressive aneurysmal shrinkage and focal neurological recovery, we did not include patients with asymptomatic, previously ruptured or treated giant cerebral aneurysms. Fusiform or serpentine-like morphology was also an exclusion criterion. A summary of the study's design is illustrated in Figure 1.

For the selected patients, we analyzed the demographics, neurological status, radiological characteristics and localization of the aneurysms, and the relevant medical history.

### Pre- and peri-procedural evaluation and endovascular procedure

The allocation of the treatment modality and the specifics of endovascular embolization were determined by a multidisciplinary working group consisting of two competent and experienced interventional radiologists and two microvascular neurosurgeons.

Neurologic and neuro-ophthalmologic evaluation was performed before treatment and at follow-up cross-sectional imaging. The modified Rankin scale was used to assess patient clinical pretreatment status and outcomes.

All patients underwent pretreatment cranial magnetic resonance imaging (MRI) and conventional digital subtraction angiography (DSA). Periprocedural three-dimensional rotational angiography (3D RA) was performed in all cases. Target aneurysm features were carefully examined, including dimensional characteristics, morphology, focal irregularities, adjacent lobulations and localization. Aneurysm volumes in mm^3^ were measured with semi-automated 3D post-processing software (MR Vessel IQ Express) in all patients. The presence of intra-aneurysmal thrombosis, parent artery luminal characteristics and their relation to the aneurysm were also considered.

Endovascular procedures were performed in patients under general anesthesia with the commercially available FD devices—the p64 flow modulation device (Phenox, Bochum, Germany) and the Pipeline embolization device (PED; Medtronic, Minneapolis, MN, USA). Technical and procedural data associated with the embolization process were gathered and analyzed. The angiographic results were evaluated with the O'Kelly-Marotta (OKM) filling grade system (19).

### Antiplatelet, anticoagulation, and anti-inflammatory medications

As part of a routine medication protocol, all patients received dual antiplatelet therapy either 75 mg clopidogrel, 2×90 mg ticagrelor or 10 mg prasugrel, and 100 mg acetylsalicylic acid daily for at least 7 days before the treatment. Every patient received a responsiveness test to clopidogrel with the VerifyNow (Accumetrics) P2Y12 assay. Levels of <70 P2Y12 response units were defined as sufficient platelet inhibition. Patients with non-response to clopidogrel or results above 70 P2Y12 response units received a substitution therapy of either 1 × 10 mg prasugrel or 2 × 90 mg ticagrelor daily. No aneurysms were treated unless substantial platelet inhibition was confirmed (20, 21). All patients received intravenous heparin intraprocedurally with an activated clotting time of >200 s. Additionally, dabigatran, 150 mg twice per day, was prescribed to every patient postoperatively to avoid rapid and severe thrombosis of the target voluminous aneurysms. Oral anticoagulation *via* dabigatran was prescribed for 8 weeks in patients with anterior circulation aneurysms and 12 weeks in patients with posterior circulation aneurysms. Intravenous application of corticosteroids was started in every patient to prevent post-therapeutic perianeurysm inflammation. Eight milligrams of dexamethasone was administered i.v. immediately after flow diverter stent placement and continued orally for the following week. Gradually, the dose was decreased by 2 mg each week. Concomitant non-steroidal anti-inflammatory drugs were prescribed for 3 weeks to maximize the prevention of aneurysmal inflammation and delayed ruptures.

### Clinical and radiological follow-up examinations

Follow-up clinical and angiographic examinations were routinely performed at 6, 12, and 24 months. Only MRI was used to evaluate the aneurysmal volume changes during follow-up. Radiological analyses and comparative studies with each prior assessment were determined on the basis of time of flight pre-contrast, 3D T1-weighted pre-contrast, time of flight (TOF) post-contrast, T2-weighted 3D CUBE, T2-weighted FLAIR 3D CUBE and 3D T1-weighted post-contrast sequences on either 1.5 or 3 Tesla scanners. Post-treatment perifocal brain parenchymal changes were assessed on T2-weighted 3D CUBE and T2-weighted FLAIR 3D CUBE sequences. All aneurysms were manually segmented by a single rater on a post-processing work station. Manual segmentation was used for 3D reconstruction, thus yielding the maximum diameter and semi-automated volume calculations for each aneurysm in each radiological examination. A decrease in aneurysmal dimensions and volume was defined as aneurysmal shrinkage.

### Statistical analysis

Data collection was performed with IBM SPSS Statistics, version 22 (Armonk, New York). The descriptive analysis presents categorical variables as percentages and absolute numbers, and continuous variables as mean and range.

## Results

### Patients' baseline characteristics

The patients' characteristics are summarized in Table 1.

**Table 1:** Baseline patient demographics and clinical presentation of the study population.

**Patients characteristic**	**Value (*n* = 36)**
Age	58.5 (range 22–76)
**Sex**	
Female (*n*)	28 (77.7%)
**Aneurysm characteristics**	
Neck (mean, mm)	8 (range 5–14 mm)
Maximal diameter (mean, mm)	26.6 (range 25–62 mm)
Pre-treatment volume (mean, mm^3^)	7,600 (range 2,225–80,822 mm^3^)
Partially thrombosed (*n*)	17
**Aneurysm localization**	
Internal carotid artery (*n*)	30
Intradural ICA	13/30
Extradural ICA	17/30
Basilar artery	4
Middle cerebral artery	2
**Clinical presentation**	
Cranial nerve III palsy	13 (36.1%)
Hydrocephaly	3 (8.3%)
Optic/chiasmal compression	6 (16.6%)
Peri-aneurysmal edema	11 (30.5%)
Other	3 (8.3%)

A total of 51 cerebral aneurysms ≥25 mm in diameter were treated in our center. Among them, 36 giant, symptomatic and previously untreated aneurysms were identified in 36 patients, 28 of whom were women (77.7%). The mean age was 58.5 years (range 22–76 years). The most common clinical presentation was impaired third cranial nerve function, which was present in 13 patients (36.1%). Hydrocephalus and cranial nerve symptoms associated with vision were documented in 8.3 and 16.6% of patients. Headache with the radiological presence of peri-aneurysmal edemic changes in adjacent brain structures was observed in 11 patients. The cohort included 36 intracranial aneurysms, 30 internal carotid aneurysms (83.3%), 4 basilar aneurysms and 2 middle cerebral artery aneurysms. Of the aneurysms across the internal carotid artery, 13 were intradural, and the remaining 17 were extradural within the proximal cavernous sinus segment of the artery. The mean maximal diameter of the treated aneurysms was 26.6 mm (range 25–62 mm), with an average neck diameter of 8 mm (range 5–14 mm). The mean luminal diameter of the target parent artery was 4.28 mm (range 3.1–5.7 mm). The mean pretreatment volume of the aneurysms was 7,660 mm^3^ (range: 2,225–80,822 mm^3^). Seventeen aneurysms showed radiological evidence of thrombus formation inside the aneurysm, defined as a >10% difference between outer and luminal volume. A total of 23 aneurysms (63.8%) were treated with sole flow diversion, and the remaining 13 were treated with loose coil packing and flow diversion in the same procedure. More extradural aneurysms were treated with sole flow diversion than with adjacent coiling. The mean maximum size of the aneurysms treated with only flow diversion appeared slightly larger (27.02 mm) than that (25.9 mm) in patients with additional coiling and flow diverter stenting.

### Radiological outcomes

Procedural and angiographic results are summarized in Table 2.

**Table 2:** Procedural and angiographic results.

**Radiological outcome**	**Early follow-up (*n*, %)**	**Intermediate 12 month**	**Long-Term 24 month**
Complete occlusion (OKM D)	23 (63.8%)	32 (89%)	31 (86.1%)
Partial occlusion or residual aneurysm (OKM B)	4 (11.2%)	–	–
Neck remnant (OKM C)	9 (25%)	4 (11%)	2 (5.5%)
Diminishing aneurysms	25 (69.4%)	30 (83.3%)	32 (88.8%)
Average volume reduction	2,108 mm^3^	3,662 mm^3^	3,917 mm^3^
Stationary aneurysms	2 (5.5%)	6 (16.6%)	1 (2.7%)
Enlarging aneurysms	9 (25%)	0	0
Complications at the point of follow-up	3 (8.3%)	0	0
Lost to follow-up	0	0	3 (8.3%)

A total of 23 patients were treated with a single flow diverter stent covering the ostium of the target aneurysm, and 13 patients required implantation of a second stent in a telescopic fashion to reconstruct the aneurysmal neck/parent vessel interface. The telescopic stenting was performed to provide the best technical execution of the embolization.

To ensure complete wall apposition and maximal coverage of the aneurysmal neck, balloon dilatation of the stent was performed in five cases. The most commonly applied stent was 5/30 mm, given the mean luminal diameter of the parent artery of 4.28 mm. We did not observe any procedural-associated technical complications regarding device migration or foreshortening of the implanted stents. The curative mechanism of flow diversion requires weeks to months to occur; therefore, the initial aneurysm occlusion rates were not substantial, as expected. Subtotal filling or OKM grade B were observed in most of the aneurysms (27; 75%); reduced perfusion with only entry remnant or OKM grade C was noted in eight (22.2%) of the aneurysms; and complete occlusion or OKM grade D was documented in only one case.

### Early radiological results

**Figure 2:**
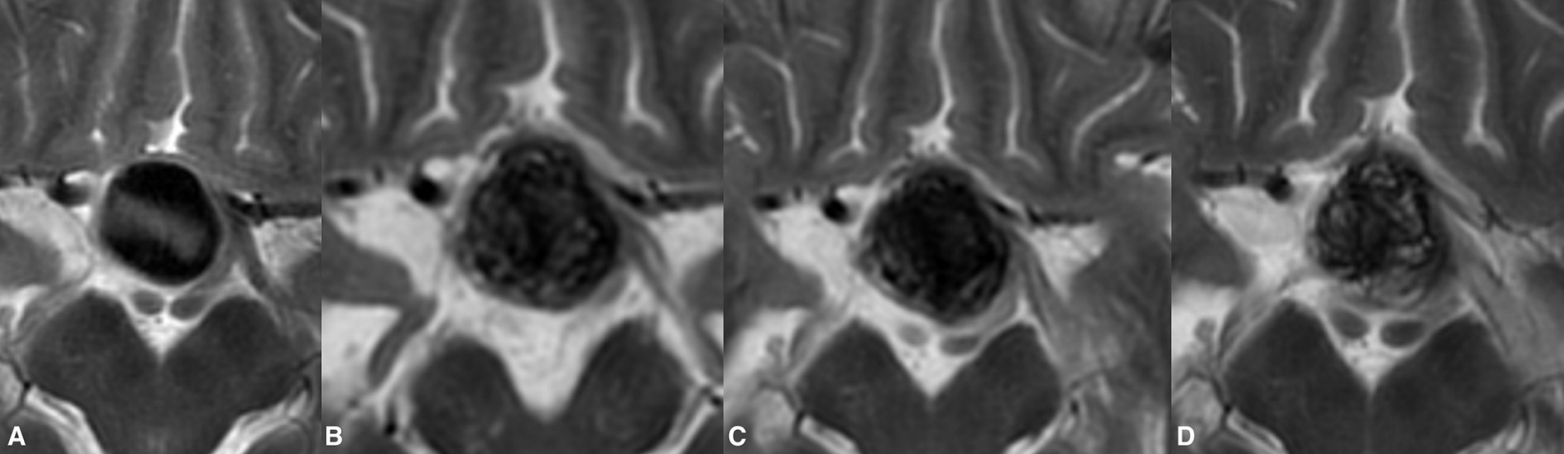
Post-treatment enlargement of a giant paraophthalmic ICA aneurysm **(A)**. During routine clinical examinations, the patient showed temporary aggravation of the presenting visual symptoms due to increase chiasmal compression. The first radiological follow-up demonstrated the treatment-related volume increase of the treated aneurysm **(B)**. Following prolonged steroid and NSID therapy, the patient's visual status improved notably. Twelve months after the treatment, the observed growth of the aneurysm was seized with documented aneurysmal shrinkage **(C)**. Lack of signal void within the aneurysm on T2WI in the same patient at 24 months indicates the ongoing aneurysm thrombosis **(D)**. The last follow-up also noted the collapse of the previous growing aneurysm.

Data from the 6 month radiological follow-up were available for all patients. Radiologically confirmed shrinkage of the treated aneurysms was confirmed in 25 cases (69.4%). The detected average volumetric reduction was 2,108 mm^3^ (mean 30.6% of the pretreatment aneurysmal volume). Negligible or no radiological changes were observed in two cases. However, post-treatment growth of the aneurysms was found in nine cases or 25% (Figure 2). The mean volumetric increase in these aneurysms was ~2,311 mm^3^. Among the extradurally located ICA aneurysms, 14 showed an average volumetric reduction of 2,080 mm^3^, whereas one remained unchanged, and two became enlarged. Six of the ICA aneurysms with intra-dural localization showed a mean 2,857 mm^3^ decrease with respect to the pretreatment volume, one remained the same, and six demonstrated an aneurysmal increase. Furthermore, during the first follow-up, aneurysms with thrombotic material present inside the lumen tended to have a slightly greater mean volume loss (2,255 mm^3^) than aneurysms without thrombi (1,972 mm^3^). Aneurysm occlusion, evaluated with catheter angiography, confirmed complete aneurysm obliteration or OKM D grade in 23 aneurysms. A progressive reduction of aneurysm perfusion in OKM grade B and grade C was observed in four and nine patients, respectively.

### Intermediate radiological results

Data for the 12 month radiological follow-up were available for all 36 patients.

Complete occlusion was found in 32 patients (89%), and a neck remnant persisted (OKM C) in four patients (11%). No signs of aneurysmal reperfusion and recanalization were confirmed. Thirty of the aneurysms showed a progressive radiological volume reduction with an average 3,662 mm^3^ loss with respect to the pretreatment volume. Either no change or negligible shrinkage was observed in the remaining six aneurysms. During this radiological follow-up, no increases in aneurysmal volume and dimensional characteristics were found.

### Long-term radiological results

**Figure 3:**
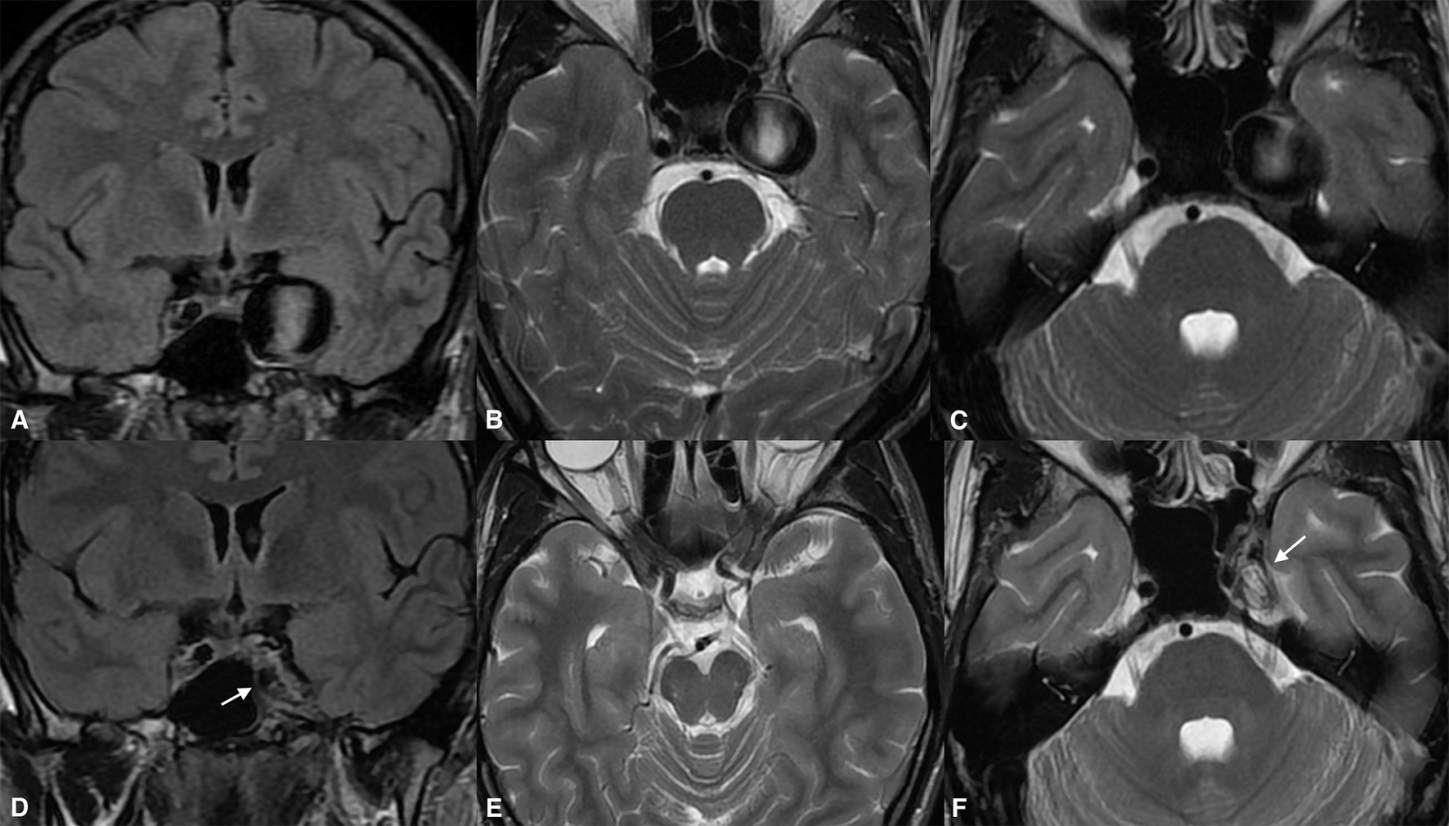
Giant cavernous internal carotid artery aneurysm causing diplopia from oculomotor nerve involvement **(A)**. The same aneurysm was causing a slight mass effect over the left temporal lobe and the adjacent brain tissue **(B,C)**. Twenty-four months after the treatment, the magnetic resonance imaging [**(D)**, arrow] demonstrated aneurysmal collapse **(E)** and elimination of the mass effect. Note the absolute shrinkage of the aneurysm with a volume reduction of up to 70% of its previous volume, as seen on the long-term follow-up [**(F)**, arrow].

**Figure 4:**
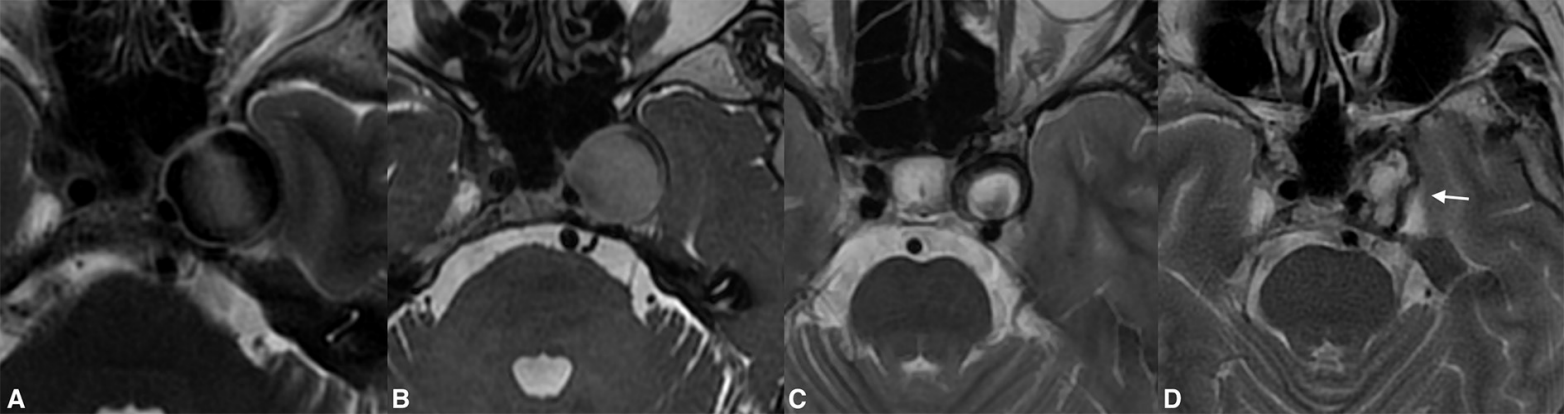
Giant extradural ICA **(A)** aneurysm demonstrating significant shrinkage following flow diversion. Despite the complete thrombosis of the aneurysm found on the first radiological follow-up, there were neglectable changes in the aneurysmal volume **(B)**. One year post-treatment, the aneurysm shrinkage was visible on the MRI **(C)**. The complete resolution of the aneurysm-induced compression of the left temporal lobe and the significantly diminished volume were present on long-term follow-up [**(D)**, arrow].

Data for the 24 month follow-up were available for 33 patients. At that point, 31 of the aneurysms had become entirely obliterated, and entry filling of the aneurysms (OKM grade C) was observed in only two cases. The MRI examinations revealed that 32 of the aneurysms demonstrated aneurysmal shrinkage, by a mean of 3,917 mm^3^ with respect to baseline (mean 47% volume loss). A comparison of results between the second and third follow-ups demonstrated that only 16 aneurysms continued to diminish (Figures 3, 4). No aneurysmal regrowth or adverse changes were found in their morphology at that point.

### Clinical outcomes

**Figure 5:**
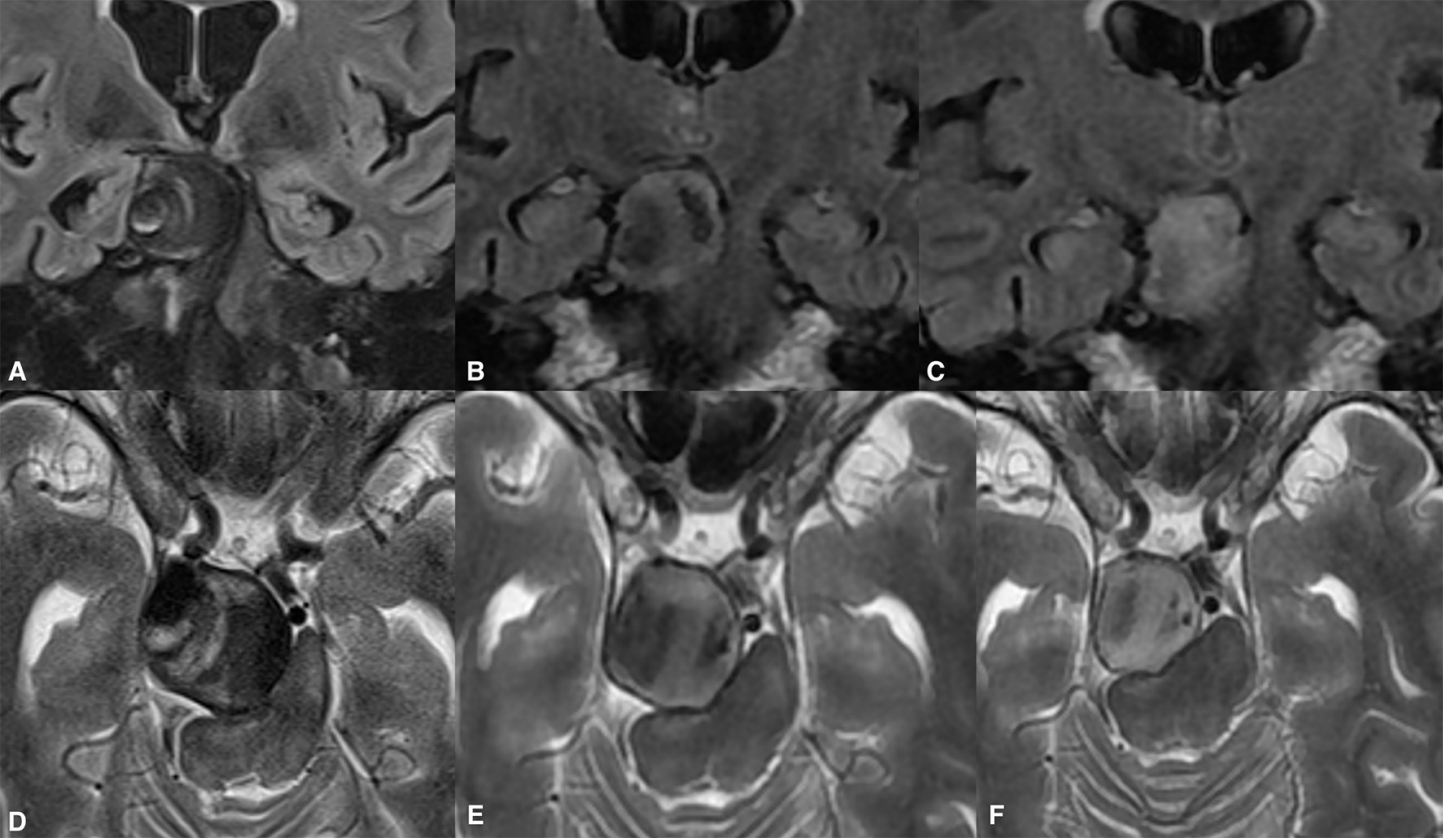
Giant partially thrombosed cerebral aneurysm causing quadriparesis and obstructive hydrocephalus due to brain stem compression **(A,D)**. Significant post-treatment clinical improvement was confirmed due to alleviation of the brain stem compression **(B,C)**. The ongoing collapse of the aneurysmal sac with progressive intrasaccular T2WI signal changes was noted during the long-term follow-up **(E,F)**.

Most of the patients demonstrated clinically significant improvements in their presenting symptoms after treatment. At the last follow-up, all 13 patients who had presented with third cranial nerve palsy showed improvements. Symptoms had resolved entirely in nine patients; one patient had minor diplopia due to persistent misalignment of the affected eye; and two had mild but ameliorated ptosis. Complete reversal of the pretreatment edematous changes was confirmed in all cases showing substantial mitigation of headaches. Quadriparesis in one patient presenting with brain stem compression and obstructive hydrocephalus was entirely resolved by the first clinical follow-up. Figure 5 provides the radiological data of the aforementioned case. Unfortunately, only two patients completely recovered from visual deficits due to optic or chiasmal compression. Among the remaining patients with presenting visual symptoms in two, vision improved from as low as finger counting to vision of 0.6.

### Treatment-related complications

Despite the aggressive corticosteroid and anti-inflammatory therapy, three patients experienced intracranial hemorrhage due to delayed rupture of their aneurysms between 1 and 4 weeks post procedurally. All three of these patients had treated aneurysms in the intra-dural segments of the internal carotid artery. Sole flow diversion was used in two of the ruptured aneurysms with loose coiling and FD stent in the remaining one. None of these complications resulted in death; however, gradual aggravation of their presenting symptoms was observed. Complete blindness was documented in the patient with the more severely ruptured aneurysm and adjacent brain hematoma. During the first clinical follow-up, two of the six patients with radiologically confirmed enlarging aneurysms showed temporary aggravation of their symptoms, thus requiring prolonged steroid and NSID therapy.

## Discussion

Cerebral aneurysms that reach a giant size (≥25 mm) without rupturing may act as space-occupying lesions (22). Compression syndromes over the locoregional neuronal tissue, dislocation or cranial nerve palsies are common clinical manifestation of these challenging lesions. The goal of the treatment is not only to prevent future hemorrhagic events but also to alleviate the associated mass effect and compression symptoms (23). Endoluminal implants that reduce and redirect the blood flow away from the aneurysm sac can diminish the pulsating phenomenon and induce steady intrasaccular thrombosis (24). Similarly to the wound healing mechanism, the biotransformation and organization of the intrasaccular thrombus to fibrous scar tissue allows the aneurysmal structure to reduce and eventually be resorbed to some extent (25). Studies have shown that this strategy effectively alleviates the compression symptoms of large and giant cerebral aneurysms (26, 27). According to Szikora et al. volume reduction and shrinkage of the aneurysm sac can be expected in most aneurysms treated with flow diversion (17). The findings of Pianto et al. are consistent with those of the abovementioned authors, and have confirmed the amelioration of mass effect symptoms after flow diversion for space-occupying aneurysms (28).

Healing and shrinkage may be preceded by acute and uncontrollable inflammatory changes inside the aneurysm that cause aggravation of compression-associated symptoms (29). Such circumstances can trigger widespread changes and potentially result in treatment-induced rupture (30). The phenomenon of delayed rupture and the triggering mechanism after endoluminal flow diversion is poorly understood. Specific conditions, i.e., hemodynamic alterations, larger sizes, complex geometry and pre-existing intrasaccular thrombosis, may trigger uncontrollable autolysis, which may overload the biologic defense mechanisms of the vessel wall and result in aneurysm rupture (31, 32). Kuzmik et al. have described the unpredictable nature of the endoluminal flow diversion, demonstrating substantially different treatment outcomes for aneurysms with the same morphology and location (33). Such complications often predispose patients to unfavorable or even fatal outcomes, because of the need for dual antithrombotic therapy with this technique (34).

This study retrospectively analyzed the volumetric changes in mass effect reduction after endoluminal flow diversion for giant cerebral aneurysms. We focused on the long-term effects of flow diversion in inducing clinical symptoms recovery and promoting aneurysmal sac collapse. Clinical improvements in impaired neurological functions are expected after the cessation of the aneurysmal pulsations followed by positive volumetric changes in the giant sac. According to Szicora et al. weeks to months are usually required before any improvement can be seen (17). In this study, at the first clinical follow-up at 6 months, 13 of 36 (36.1%) patients with CN III palsy who underwent treatment for their giant aneurysms demonstrated significant amelioration of their presenting symptoms. First, MRI radiological follow-up surveillance at 6 months confirmed shrinkage of the treated aneurysms in 25 cases (69.4%). The average volume mass reduction of 2,108 mm^3^ induced by the treatment clearly facilitated the clinical recovery of the analyzed patients. Our mid- and long term results suggest that this method induces a steady but progressive disintegrative volume effect. The available MRI follow-up imaging revealed that aneurysmal collapse and mass effect resolution continued up to 24 months after the treatment. Importantly, we emphasize that, in our cohort, the greatest decrease in mass occurred within the first 6 months and in aneurysms with thrombotic changes inside the sac. Although the comparison was not statistically proven, in our study, aneurysms treated with sole flow diversion tend to have a better shrinkage rate than aneurysms that underwent coil implantation.

Moreover, we demonstrated that the locations of target giant aneurysms might predict the posttreatment behavior. The extradural ICA aneurysms treated in our cohort were more likely to decrease in size than intradurally located aneurysms. Similar findings and expectations have been suggested by Carneiro et al., according to their experience with FD and extremely large and giant intracranial aneurysms (35). Transient sac increase after flow-diversion for giant aneurysms is a well-recognized scenario (17, 27, 36, 37). Post-treatment, substantial aneurysmal enlargement in our cohort occurred in nine patients. At the second follow-up at 12 months, all the previously enlarging aneurysms demonstrated a slow collapse with mixed T1WI-T2WI signal intensity. Because these changes were observed only during the early follow-up, we believe they are probably part of the rapid evolution of the blood clots (38).

Although our study conceptualization and applied methods were not aimed at analyzing and discussing the angiographic occlusion results, the observed obliteration rates were consistent with those reported in the available FD literature (39–43). Our angiographic results suggested the unequivocal effect of flow diversion on giant aneurysms; however, the complications associated with this approach should not be overlooked. Although no patients died in this study, we documented unfavorable outcomes due to post-procedural aneurysmal rupture or worsened clinical symptoms due to aneurysmal enlargement. The post-procedural aneurysmal rupture documented in our series occurred in three patients, in the first month after the conduced stent implantation, thus resulting in an 8.3% procedural-associated hemorrhagic complication rate. A similar rate of 7% early rupture of aneurysms treated with FDs has been found by Cagnazzo et al. who have highlighted that such events might be more likely to occur with sole flow-diversion than flow-diversion and adjacent coiling of the target aneurysm (2). Similarly, Lee et al. have suggested that sole flow diversion in large cerebral aneurysms might be associated with increased hemorrhagic rates (27). Interestingly, in our series, two of the three patients that experienced delayed rupture of their aneurysm were treated with sole flow diversion. However, we stray further from conclusions about whether coiling can mitigate delayed complications or induce them. This is mainly because the decision to use coils or not is based on the main operator discrepancy, resulting in a reliable source of bias.

To date, no consensus or specific guidelines have been published regarding the therapeutic management of these patients. The unpredictable and uncontrollable post-treatment inflammatory process inside aneurysms is likely to be associated with both the curative and the harmful mechanisms (44). Published neuro-interventional data regarding corticosteroid and anti-inflammatory drug management are inconsistent (45, 46). Corticosteroids are well-known to play an essential role in the modulation of the inflammatory response. These medications are effective in controlling cerebral vasogenic edema. Corticosteroids specifically inhibit platelet adhesion, thrombus formation and platelets' interactions with monocytes. In combination with the anti-inflammatory effects of non-steroidal anti-inflammatory drugs and the oral anticoagulant treatment, this therapy was our post-procedural management protocol for every patient (47). We believe that it could theoretically alleviate the autolysis of the aneurysm by controlling the thrombotic transformation and the ongoing inflammation. Therefore, we believe that the mitigation of the processes associated with aneurysmal thrombosis was responsible for the diminished volume at the first follow-up.

This study has several limitations, mainly due to its retrospective nature and small cohort. Our individual experience limited tailoring of the technical results and strategy. Furthermore, the lack of blinding might be interpreted as a source of bias. Therefore, our study results should be interpreted with caution and may not be widely applicable. Furthermore, we acknowledge that most of the aneurysms were located across similar locations, thus potentially influencing our results and observations.

## Conclusion

In this small series, we demonstrated that favorable volumetric reduction and aneurysmal sac shrinkage were obtained with endovascular flow diversion. Thus, radiologically confirmed reversal of the mass effect and subsequent clinical improvement can be expected in most cases. Progressive aneurysmal collapse is a time-consuming process requiring long-term follow-up. Finally, treatment-associated complications should be considered for flow diversion in patients with giant cerebral aneurysms.

## Data availability statement

The raw data supporting the conclusions of this article will be made available by the authors, upon reasonable request.

## Ethics statement

Ethical review and approval was not required for the study on human participants in accordance with the local legislation and institutional requirements. The patients/participants provided their written informed consent to participate in this study.

## Author contributions

KS and SS: writing. KS, SM, and MP: verifying and secondary data analysis. KM, KN, AH, II, and VK: conceptualization of the study and critical review. All authors contributed to the article and approved the submitted version.

## Conflict of interest

The authors declare that the research was conducted in the absence of any commercial or financial relationships that could be construed as a potential conflict of interest.

## Publisher's note

All claims expressed in this article are solely those of the authors and do not necessarily represent those of their affiliated organizations, or those of the publisher, the editors and the reviewers. Any product that may be evaluated in this article, or claim that may be made by its manufacturer, is not guaranteed or endorsed by the publisher.
